# Enhancing Physician Managerial Capabilities: Partnership between Medicine and Business

**DOI:** 10.15694/mep.2019.000096.1

**Published:** 2019-04-25

**Authors:** Samuel Siu, Andrew D. Scarffe, David R. Barrett, Michael J. Strong, Valerie Schulz, David R. Dixon, James E. Calvin

**Affiliations:** 1 Schulich School of Medicine and Dentistry; 2 Ivey International Centre for Health Innovation; 3 Office of the President; 4 Continuing Professional Development

**Keywords:** Physician leadership, management principles, business principles

## Abstract

This article was migrated. The article was marked as recommended.

Background

Physicians are typically appointed to leadership roles within health care organizations on the basis of individual accomplishments in research, education, and/or clinical care. However, these types of achievements seldom provide the requisite management capabilities to lead within health organizations. In this manuscript, we described our initial experience in developing an in-house program to provide current and aspiring physician leaders with the managerial capabilities to enhance the quality of health care delivery within their respective organization.

Methods

In a partnership established between a Medical School and a Business School, we designed two series of weekend workshops to provide current and aspiring physician leaders with the financial capabilities to assist them in their future healthcare leadership careers. This course was then expanded to a Management Principles for Physician workshop with open enrollment to physicians at all levels. Baseline demographics and participant evaluations of each course were recorded. In the open enrollment Management Principles for Physician workshop, we examined the relationship between participant background and their course evaluations as well as their areas of interest for further training.

Results

All 3 workshops received excellent evaluations by participants. The positive impact of the open enrollment program, based on participants’ self-evaluations, was the highest in female physicians, as well as early to mid-career physicians. Additionally, physicians who do not currently hold leadership positions and those who are leading at Divisional levels were the most interested in further training in finance.

Conclusion

In summary, this series of workshops demonstrated the feasibility of an in-house physician leadership program and yielded important information for the design of future leadership development curriculum.

## Introduction

Physicians are typically appointed to leadership roles within health care organizations on the basis of sustained and exemplary individual accomplishments in research, education, and/or clinical care. However, these past achievements seldom provide the requisite capabilities necessary to lead within health organizations with their complex reporting relationships, multiple stakeholder networks, and the prevailing collaborative approach to setting and implementing strategic and operational goals. ( Stoller, 2009; Arroliga *et al.*, 2014) Many physician leaders acquired their managerial skills on the job, the so called “accidental” leaders. ( Ackerly *et al.*, 2011) Physician leaders are increasingly aware that they need managerial training to fulfill their responsibilities to enhance the quality of health care delivery. ( Jaffe *et al.*, 2016) Some of the learning needs identified by physician leaders include: 1) financial planning, 2) conflict resolution and negotiation, 3) communication, 4) strategic analysis. ( Gagliano *et al.*, 2010)

In recognition of the need for managerial acumen in physician leaders, several organizations have created their own in-house physician leadership training. ( Tangalos *et al.*, 1998; Stoller, Berkowitz and Bailin, 2007; Cherry, Davis and Thorndyke, 2010; Murdock and Brammer, 2011; Bircher, 2013; Clyne, Rapoza and George, 2015) Additionally, Medical associations and societies have also established physician leadership programs, with recognitions for completing of set of courses with demonstration of leadership accomplishments. ( AAPL, 2018; Joule, 2018; Rotman, 2018) These physician leadership programs can limit participation by being targeted towards physicians who are already in leadership positions, and frequently require a significant time commitment for completion. With their clinical and/or academic responsibilities, physician leaders may not have the time or the resources for such a longitudinal program.

This paper describes our initial experience in developing an in-house program to enhance the managerial capabilities of physicians that can be applied to the delivery of quality health care.

## Methods

Our strategy was to provide fundamentals in managerial capabilities for physician leaders, in a series of courses using a case based approach, delivered in an interactive setting, and in a workshop format that recognizes limited time and financial resources faced by physician leaders. This high intensity, condensed program was mindfully developed to provide a broad exposure to managerial fundamentals and was not designed to take the place of more extensive courses, such as Master of Health Administration or Master of Business Administration programs. While we anticipated that most participants would be current or aspiring physician leaders from academic centers, the curriculum was applicable to physicians at all career stages from both academic and community organizations.

The program evolved through several stages. The first offering was motivated by the Department of Medicine (DOM) requirement that a business plan should accompany a proposal for new programs submitted by Divisional Chairs. This business plan is intended to provide the basis for assessing the quality dimension (outcomes/cost) of the proposed program. In consultation with the sponsoring Department of Medicine Chair/Chief (JEC), a physician leader with an executive Master Business Administration (SCS) created a template for a workshop in financial analysis and planning and then co-developed the curriculum with members of the Ivey International Centre for Health Innovation at the Ivey Business School (ADS, DRB). The initial workshop was held on two consecutive Saturdays in January 2017 and delivered by Ivey business school faculty with experience in health care sector. The participants were members of the Department of Medicine executive committee, comprised of Chair of Medicine, Vice Chairs, Divisional Chairs, and the Residency Training Program Director. Using an interactive case based format, the participants were introduced to fundamentals of: 1) Hospital administration and not-for-profit management; 2) Strategic business development; 3) Financial statement analysis; 4) Financial modeling using Excel; and, 5) Business case development (see Table 1). As part of accreditation for continual medical education credits, all participants completed a program evaluation which also served as feedback for modification of future curriculum.

Based on the quantitative course evaluations and qualitative comments from participants of the January workshop, a follow up program on financial analysis and planning was developed, again sponsored by the Department of Medicine Chair. This second workshop provided a more in depth exposure to financial planning and also provided a review of the initial workshop fundamentals for those participants that were not part of the first workshop. Participants in this second workshop included Department of Medicine executive committee members, and also a select group of current or emerging Department of Medicine physician leaders. The second workshop was held over two consecutive weekend days in December 2017 (see Table 1).

At the end of both finance workshops, participants were asked to reflect on the program and identify areas for future curriculum development. Based on the spectrum of responses, we designed a course that incorporated general managerial principles (i.e., Management Principles for Physicians) and conducted the inaugural course in March 2018 (see Table 1). In contrast to the two prior financial workshops, this course was open-enrollment and each participant was responsible for the course fee which was set at a cost recovery basis. A steering committee (SCS, VS, DRD) associated with the Medical School were created to insure that the course content and design met accreditation requirements for general and specialty physicians. The course curriculum received executive input from the Dean of the Medical School (MJS) and Department of Medicine Chair (JEC).

Baseline demographics of the participants (i.e., years of independent practice, academic rank, nature of leadership position, and site of practice) were collected via publicly accessible university, hospital, or provincial licensure websites. At the end of each course, participants were asked to anonymously complete evaluations as to whether the course enhanced their knowledge base, benefited their role, or was relevant to their role on a 5 point Likert scale (i.e., strongly disagree, disagree, neutral, agree, strongly agree). For the Management Principles for Physician course (as part of needs assessment for future course offerings) participants were asked to provide their level of leadership (i.e., none, division, department, organization wide [such as hospital or region]), whether they had enrolled in prior physician leadership courses, whether the course format is feasible for future offerings, potential barriers to future enrollment, and what should be the focus on future course offerings.

As the goal of these workshops was to enhance the managerial capabilities of physician leaders in order to improve efficiency, effectiveness and quality of health care delivery, the programs are considered to be quality improvement initiatives and do not require research ethics approval for reporting of results. ( Research, 2017)

Counts, medians with Interquartile range, or proportions were provided whenever appropriate. Characteristics of participants enrolled in the three workshops were compared. Univariate analyses were performed to determine relationships between participant characteristics and evaluation scores. Additional evaluations were performed to examine relationship between participant characteristics and request for focus of future courses. Comparisons were performed using Fisher’s exact or non-parametric Kruskall-Wallis tests whenever appropriate. Two sided level of significance was set at a P-value of <0.05. All statistical analysis was performed by SPSS for Windows Version 24 (Armonk, NY:IBM Corp).

## Results/Analysis

Baseline demographics of the three courses are summarized on Table 2. As workshop enrollment was expanded from Department of Medicine executive committee members (Finances for Physician Leaders 1) to Department of Medicine Physician Leaders (Finances for Physician Leaders 2), to open enrollment (Management Principles for Physicians), there is a reduction in median years of independent practice, greater range in academic rank, nature of leadership role, and site of practice. Importantly, half of the participants in the open enrollment course were early career or in community practice. The proportion of female participants was the highest in the open enrollment course. Four of the 11 female participants (36%), and 8 of 13 male participants (64%) were associate professor or professor.

**Figure 1.**
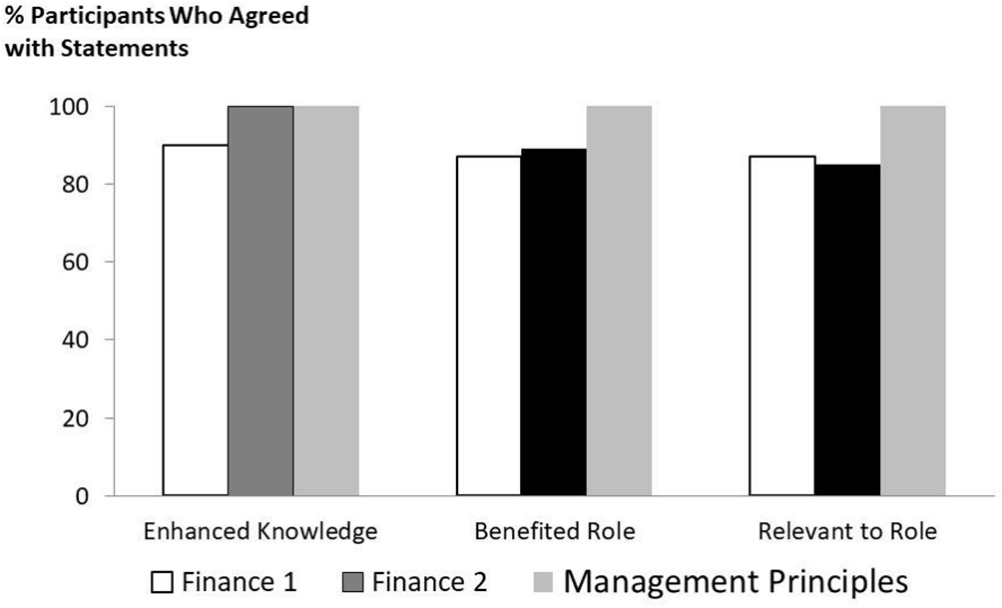
Proportion of participants that indicated that they agreed or strongly agreed with each of the 3 statements: 1) At the completion of the course, their knowledge was enhances; 2) At the completion of the course, the new knowledge will benefit their role or practice; 3) The course curriculum was relevant to their role or practice

Sixteen of 19 participants (84%) completed evaluations after Finances for Physician Leaders I workshop while 25 participants (86%) completed evaluations after Finances for Physician Leaders II workshop. Between 85 and 100% of participants who completed evaluations in the two finances courses reported enhancement of knowledge, benefits of course material to their practice or role, or relevance of course material to their practice or role ( Figure 1). In contrast, those 23 participants (96%) who attended the Management Principles for Physician course and completed their evaluations, were unanimous in agreeing or strongly agreeing that their knowledge was enhanced and that the course curriculum was beneficial and relevant to their roles. The majority of early to mid-career participants (i.e., year of practice 1-10 years) gave the course the highest rating (i.e., strongly agree) in the enhancing knowledge ( Table 3). Female participants were more likely to highly agree that their knowledge was enhanced and that course material were relevant to their role ( Table 3). Prior participation in physician leadership programs, site of leadership, level of leadership were not significantly related to evaluation scores for any of the 3 categories of enhancing knowledge, benefit to role, and relevance to roles.

**Figure 2.**
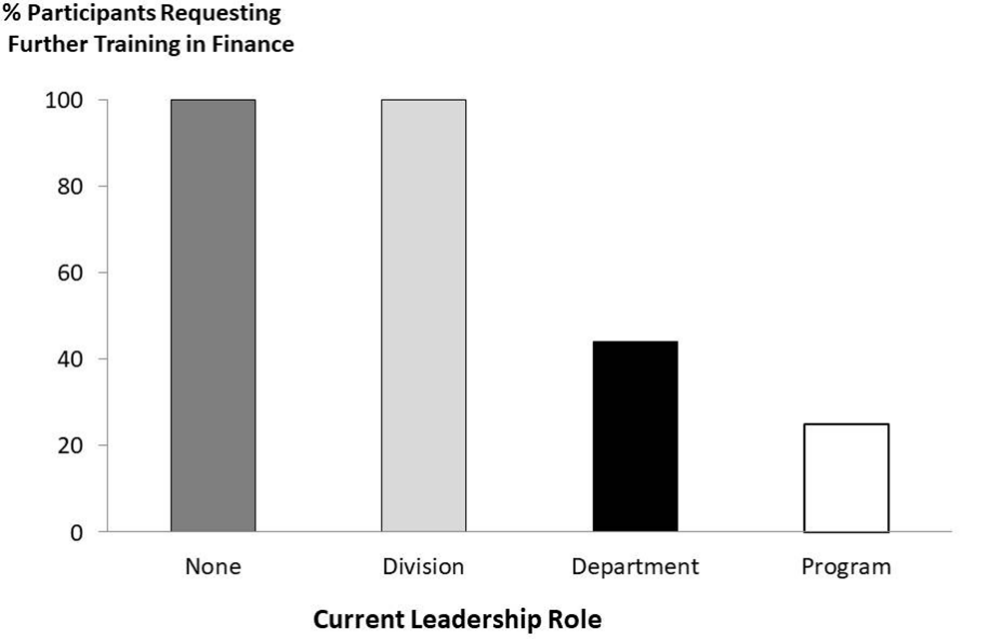
Proportion of participants who requested financial training in future course offerings

Twenty-two participants (96%) from the Management Principles for Physicians workshop were interested in follow up courses using a similar format. When asked as to the focus of future course offering, >50% of participants identified financial modeling (65% of responses), leading and organizational behavior (61% of responses), and value based health care (52%) as additional areas of interest. Those without a formal leadership role, or whose roles were at Divisional levels, were more likely to express interest in finance than those who hold leadership positions at Departmental, Enterprise or Programmatic levels (p=0.02) ( Figure 2).

**Table 1.** Curriculum Summary for the Three Courses

Finance for Physician Leaders 1	Finances for Physician Leaders II	Management Principles for Physicians
Not-for-Profit Management	Analytics for Physicians	Leading and Organizational Behavior
Managing Financial Resources	Decision Logic	Value base Health Care
Capital Budget Planning & Financial Statements	Health System Organization and Financing	Analytics for Financial Planning
Introductory Analytics	How to start a new program	Managerial Accounting
Application of Analytics for Health Care	Acquisition or Recruitment of a new position	Business Plan Development
Business Case Development and Assessment	Evaluating an Existing Program	Operational Management

**Table 2.** Characteristics of Participants

	Finances for Physician Leaders I N (%)	Finances for Physician Leaders 2 N (%)	Management Principles for Physicians N (%)	P
Number of participants	19	29	24	
Female	5 (27%)	10 (35%)	11 (46%)	0.30
Years in Independent Practice (median, Inter quartile range)	21 (14-31)	15 (6-23)	10 (2-21)	**0.001**
Academic Rank None Assistant Professor Associate Professor Professor	3 (16%) 9 (47%) 7 (37%)	10 (35%) 11 (38%) 8 (27%)	6 (25%) 6 (25%) 5 (21%) 7 (29%)	**0.023**
Nature of Leadership Position Enterprise/Regional Departmental Divisional None	- 5 (26%) 14 (74%) -	- 5 (17%) 17 (59%) 7 (24%)	1 (4%) 5 (20%) 9 (38%) 9 (38%)	**0.006**
Site of Practice University Hospital Community	19 (100%)	29 (100%)	18 (75%) 6 (25%)	**0.003**

**Table 3.** Association between Participant Characteristics and the Highest Rating Score as to the Benefits or Relevance of the Management Principles for Physicians Course

	Participant Characteristic	N (% of total in category)	P value
Strongly agreed that Knowledge was Enhanced	Career stage: 1-5 years 6-10 years >10 years	6 (85%) 8 (80%) 1 (17%)	**0.016**
Strongly agreed that Knowledge was Enhanced	Female Male	10 (91%) 5 (42%)	**0.027**
Strongly agreed that Course was Relevant to Role	Female Male	7 (64%) 2 (17%)	**0.036**

## Discussion

We have demonstrated the feasibility of an in-house physician leadership program, first focusing on enhancing financial capabilities and then expanding the curriculum to other managerial areas. We found that the impact, as based on participant’s evaluations, was the highest in physicians who identified as female, as well as early to mid-career physicians. Additionally, physicians who do not currently hold formal leadership roles (i.e., aspiring leaders) and those who are leading at Divisional levels expressed the highest interest in further exposure to financial concepts and practices.

There are previous published reports of in-house physician leadership programs either sponsored by physician societies or hospitals. ( Tangalos *et al.*, 1998; Stoller, Berkowitz and Bailin, 2007; Cherry, Davis and Thorndyke, 2010; Murdock and Brammer, 2011; Bircher, 2013; Agius *et al.*, 2015; Clyne, Rapoza and George, 2015) Most of these programs are restricted to either physicians who are already appointed to leadership position or have been identified to be potential leaders. ( Bachrach, 1997; Tangalos *et al.*, 1998; Stoller, Berkowitz and Bailin, 2007; Clyne, Rapoza and George, 2015) While this approach concentrates resources on those individuals who would be best positioned to apply their new capabilities to the immediate benefit of their respective organizations, there is a risk that early career physicians may be excluded as they have not had the exposure to the decision makers to be considered for leadership development. Early career physicians represent a group that may offer the best return on investment as they will have a longer period of time to apply their managerial capabilities to enhance quality of care compared to their more senior colleagues. As can be seen in our open enrollment program, 25% of our participants were early career (<5 years) and 25% were mid-career (6-10 years), demonstrating that interest in managerial principles spans all career duration. As physicians frequently practice within health systems with complex reporting relationships, and where quality of health care delivery is a key metric for funding, it is important for all physicians working in health systems to have a foundation in management sciences. ( Ivers, Dhalla and Brown, 2018) Some have suggested that physicians in training should be exposed to managerial sciences, ( Arroliga *et al.*, 2014; Block, 2014), with preliminary reports of leadership curriculum being incorporated into medical school and specialty training programs to increase physician leadership capabilities so as to enhance team functioning and the quality of care. ( Bircher, 2013; Clyne, Rapoza and George, 2015)

It would be unrealistic to expect that we can provide the managerial skills required to be an effective physician leader with a two-day course. We do expect that our courses will provide foundational capability and enable the physician attendee to have more informed dialogue with their administrative counterparts. Our two day format was well received and provided enough depth for an effective learning experience. As the evaluations of these inaugural courses were highly favorable, and there is high level of interest for future offerings, we see these courses as a template for a modular approach to physician leadership training. A modular approach would enable future offerings to be customized along specific streams which will be tailored to individual’s specific topical interest and level of leadership responsibilities.

We observed that early to mid-career physicians had the most interest in follow up courses in finance, likely relating to a desire to be prepared for future responsibilities with budgets and quality of care. In contrast, more senior physician leaders may have had some exposure to finance and have teams with financial expertise, and are more interested in strategic issues like value based health care. Another approach to the open enrollment is that access to rural-based physicians, who may be appointed to leadership position within their community, but may not have the same access to mentors and infrastructure of a tertiary care center. A physician leadership curriculum that is relevant for community physicians would be a model by which an academic health center can enhance the quality of health care delivery beyond its catchment area. ( Murdock and Brammer, 2011)

An open enrollment program would reduce inherent or hidden biases in promotion and advancement in any organization, and may provide a more grounded bottom-up approach to changing organizational culture. ( Bachrach, 1996; Snyderman, Eibling and Johnson, 2011) This phenomenon is exemplified by the issue of gender differences in academic promotion;female faculty was less likely to achieve the rank of associate professor or full professor in comparison to their male counterparts. ( Tesch *et al.*, 1995; Nonnemaker, 2000; Carnes, Morrissey and Geller, 2008) Possible explanations for this disparity include difference in academic resources, administrative roles, and negotiation skills. ( Carr *et al.*, 1993; Tesch *et al.*, 1995; Nonnemaker, 2000; Tomer *et al.*, 2015) The observation that female faculty being provided with fewer institutional resources has been described as a “sticky floor” as opposed to a “glass ceiling” ( Tesch and Nattinger, 1997; Carnes, Morrissey and Geller, 2008). It is for this reason that open enrollment in the program is essential to providing females and males with equal opportunity to access formalized programming and resources that will enhance their managerial capabilities. Indeed, in our open enrollment program, there was equal proportion of female and male participants. Interestingly, approximately 1/3 of female participants were at associate or full professor rank, in contrast to 2/3 of the male participants. Irrespective of gender, physician leadership training may provide a new path for career advancement or academic promotion for early and mid-career physicians. ( Fairchild *et al.*, 2004; Andrews, 2007)

The rationale to have the Ivey business school faculty deliver our curriculum was based on several factors. The curriculum content falls within the area of expertise of faculty who are found within a business school and the instructors are experienced and trained in case-based teaching for learners at all levels. Additionally, all of the Ivey faculty in our program had either expertise within the health care space as consultants, collaborators, or researchers, thus they would have both the expertise, experience, and the credibility to engage a highly accomplished and discerning physician audience. Their familiarity of the local health system also brought relevance to the concepts that were being taught. ( Gagliano *et al.*, 2010)

We are likely delivering the curriculum to a highly motivated subgroup that may have been already selected or self-selected. However, the weekend and modular approach should minimize barriers. Indeed, in the post course survey when participants were asked whether there were barriers to participation in future courses, none stated cost as being a barrier to enrollment. In any quality improvement initiative, an intervention should be followed by measurement of an improvement in outcome. For the purpose of this report, we are documenting participant evaluations as a short term surrogate for outcome, while acknowledging that longitudinal follow up will be necessary to demonstrate a definitive benefit. However we are encouraged by reports that a leadership training program for female physicians reported higher ranking in leadership attributes in the 4 years after completion of the program, as compared to those who were not accepted or a general population of women physician. ( Dannels *et al.*, 2008) Another study reported that positive outcomes of their physician leadership program as the number of business plans either implemented or deferred over a 14 year period. ( Stoller, Berkowitz and Bailin, 2007)

## Conclusion

In summary, we demonstrated the feasibility of an in-house physician leadership program jointly developed by a medical school and a business school. Importantly, the positive impact of the open enrollment program received the highest rating from female physicians as well as early to mid-career physicians. Additionally, physicians who do not hold currently leadership positions and those who are leading at Divisional levels were the most interested in further training in finance. These preliminary findings will be important considerations in the design of future physician leadership programs.

## Take Home Messages

• Physician leadership program, jointly developed by medical and business schools, can be effectively delivered via a weekend workshop format.
• The positive impact of our open enrollment physician leadership program received the highest rating from female physicians as well as early to mid-career physicians.
• Physicians who do not hold currently leadership positions and those who are leading at Divisional levels were the most interested in further training in finance.

## Notes On Contributors

SCS is Professor of Medicine and Faculty Coordinator for Physician Leadership Program, Schulich School of Medicine and Dentistry, Western University, London, Ontario, Canada, https://orcid.org/0000-0002-1104-3669

ADS is Research Associate, Ivey International Centre for Health Innovation, Ivey Business School, Western University, London, Ontario, Canada.

DRB is Assistant Professor of Operational Management and Executive Director, Ivey International Centre for Health Innovation, Ivey Business School, Western University, London, Ontario, Canada.

MJS is President of the Canadian Institutes of Health Research, Ottawa, Canada, and Immediate Past Dean of Schulich School of Medicine and Dentistry, Western University, London, Ontario, Canada.

VS is Associate Dean, Continuing Professional Development, Schulich School of Medicine and Dentistry, Western University, London, Ontario, Canada.

DRD is Senior Advisor, Continuing Professional Development, Schulich School of Medicine and Dentistry, Western University, London, Ontario, Canada.

JEC is Professor and Chief/Chair of Medicine, Schulich School of Medicine and Dentistry, Western University, London, Ontario, Canada.

## Declarations

The author has declared that there are no conflicts of interest.

## Ethics Statement

As the goal of these workshops was to enhance the managerial capabilities of physician leaders in order to improve efficiency, effectiveness and quality of health care delivery, the programs are considered to be quality improvement initiatives and do not require research ethics approval for reporting of results. (Research Western University. Distinguishing Between Quality Assurance/Improvement, Program Evaluation & Research. 2017)

## External Funding

This article has not had any External Funding
