# Supplementary material for: Enhancing Physician Managerial Capabilities: Partnership between Medicine and Business
Source: MedEdPublish (2016). 2019 Apr 25;8:96. [Version 1] doi: 10.15694/mep.2019.000096.1 (PMC10712598; doi:10.15694/mep.2019.000096.1)
Supplement: Peer review history

# Reviewer report: Reviewer response for version 1

**DOI:** 10.21956/mep.19839.r29916

**Published:** 2019-05-08

**Authors:** Trevor Gibbs

**Affiliations:** 1 AMEE

This review has been migrated. The reviewer awarded 4 stars out of 5 An interesting paper that covers a very important topic - the amount of leadership / management training that physicians need. I felt that this was a very well written paper, very clear and appropriate. My only slight criticism is the lack of longer term evaluation.

Reviewer Expertise:

NA

No decision status is available

# Reviewer report: Reviewer response for version 1

**DOI:** 10.21956/mep.19839.r29915

**Published:** 2019-04-25

**Authors:** P Ravi Shankar

**Affiliations:** 1 American International Medical University

This review has been migrated. The reviewer awarded 4 stars out of 5 I enjoyed reading this interesting manuscript. Management and leadership skills have not received enough attention and doctors often find themselves taking up managerial and leadership roles without proper training. Many educational fellowship and other programs do offer leadership training but training in finance is more difficult to obtain. The authors have described their in house educational program in detail. All medical educators will find this article to be of interest.

Reviewer Expertise:

NA

No decision status is available
